# Cryptic *FUS-ERG* fusion identified by RNA-sequencing in childhood acute myeloid leukemia

**DOI:** 10.3892/or.2013.2751

**Published:** 2013-09-25

**Authors:** IOANNIS PANAGOPOULOS, LUDMILA GORUNOVA, BERNWARD ZELLER, ANNE TIERENS, SVERRE HEIM

**Affiliations:** 1 Section for Cancer Cytogenetics, Institute for Medical Informatics, The Norwegian Radium Hospital, Oslo University Hospital, Oslo, Norway; 2 Centre for Cancer Biomedicine, Faculty of Medicine, University of Oslo, Oslo, Norway; 3 Department of Pediatric Medicine, Oslo University Hospital, Oslo, Norway; 4 Department of Pathology, The Norwegian Radium Hospital, Oslo University Hospital, Oslo, Norway; 5 Faculty of Medicine, University of Oslo, Oslo, Norway

**Keywords:** acute myeloid leukemia, RNA-sequencing, *FUS*, *ERG*, fusion gene

## Abstract

Sequential combination of cytogenetics and RNA-sequencing (RNA-Seq) has been shown to be an efficient approach to detect pathogenetically important fusion genes in neoplasms carrying only one or a few chromosomal rearrangements. We performed RNA-Seq on an acute myeloid leukemia in a 2-year-old girl with the karyotype 46,XX,add(1)(p36), der(2)t(2;3)(q21;q21),del(3)(q21),der(10)t(1;10)(q32;q24),der(16)(2qter-->2q21::16p11-->16q24::16p11-->16pter)[13]/46,XX[2] and identified a cryptic *FUS/ERG* fusion gene. PCR and direct sequencing verified the presence of the *FUS-ERG* chimeric transcript in which exon 7 of *FUS* from 16p11 (nt 904 in sequence with accession number NM_004960 version 3) was fused in frame to exon 8 of *ERG* from sub-band 21q22.2 (nt 967 in NM_004449 version 4). The *FUS-ERG* transcript found here has been reported in only two other cases of childhood leukemia, in a 1-year-old boy and an 8-month-old boy, both diagnosed with precursor B cell ALL. The fusion transcript codes for a 497 amino acid residues FUS-ERG protein and, similar to other AML-related FUS-ERG fusion proteins, contains both functional domains (TR1 and TR2) of the transactivation domain of FUS and the ETS domain of ERG. The clinical significance, if any, of the amino acid residues which are coded by the exons 8, 9 and 10 of *ERG* in the fusion FUS-ERG proteins, remains unclear.

## Introduction

The *FUS/ERG* fusion gene (*FUS* is also named *TLS*) was first described in 1994 by two different groups. Ichikawa *et al*(1) found the *FUS/ERG* in four acute myeloid leukemia (AML) patients whereas Panagopoulos *et al*(2) identified *FUS/ERG* in bone marrow cells carrying a t(16;21)(p11;q22) in a 3-year-old boy diagnosed with AML M1. Since then, the t(16;21)(p11;22) and/or its fusion product *FUS/ERG* has been reported in 66 cases of AML and 3 cases of acute lymphoblastic leukemia (ALL; http://cgap.nci.nih.gov/Chromosomes/Mitelman, database updated May 15, 2013). It is seen in all ages and appears to be associated with a dismal prognosis (3,4). Nevertheless, survivals longer than 36 months have been reported in 3 childhood cases one of which was an ALL (4–6). The same fusion *FUS/ERG* has also been found in 3 Ewing tumors (8,9); it is thus one of the relatively few fusion genes that exert pathogenetic influence in widely disparate neoplastic entities.

Pereira *et al*(7) showed that retroviral transduction of *FUS/ERG* to human umbilical cord blood cells altered myeloid and arrested erythroid differentiation and led to a dramatic increase in the proliferative and self-renewal capacity of transduced myeloid progenitors. They concluded that ‘*TLS-ERG* expression alone induced a leukemogenic program that exhibited similarities to the human disease associated with this translocation’. Zou *et al*(10) showed that *FUS/ERG* activated different sets of genes in mouse L-G myeloid progenitor cells and NIH3T3 fibroblasts; the two cell types show little similarity. They concluded that FUS/ERG transformed hematopoietic cells and fibroblasts via different pathways.

In the two original articles, *FUS/ERG* was identified based on the candidate gene approach (1,2). Prior to those two studies, the breakpoints of the t(16;21)(p11;q22) had already been shown to cluster in a specific intron of *ERG* in chromosome band 21q22 (11). *ERG* was furthermore known to be fused with *EWSR1* (on 22q12) in a subset of Ewing sarcomas (12,13), and *EWSR1* was known to display a high degree of homology with *FUS*, the 16p11 gene rearranged in the t(12;16)(q13;p11) that characterizes myxoid liposarcomas (14,15). It was therefore decided to investigate whether *FUS* was also involved in the t(16;21) of AML. In both studies, Southern blot analysis demonstrated that *FUS* was rearranged and PCR examinations showed the formation of a *FUS/ERG* fusion gene (1,2).

Recently, RNA-sequencing (RNA-Seq, also known as whole transcriptome sequencing) was shown to be an efficient tool in the detection of fusion genes in cancer (16). However, it suffers from the shortcoming of identifying as ‘fusion genes’ also many technical, biological and, perhaps in particular, clinical ‘false positives’, thus making the assessment of which fusions are important and which are noise extremely difficult. We and others have shown that a combination of cytogenetics and RNA-Seq is an excellent approach to detect the ‘primary’ fusion genes in neoplasms carrying only one or a few chromosomal rearrangements. In solid tumors, this approach was used to identify the *WWTR1-CAMTA1* and *YWHAE-FAM22A/B* chimeric genes in epithelioid hemangioendothelioma and in high-grade endometrial stromal sarcomas, respectively (17,18), *ZC3H7-BCOR* in endometrial stromal sarcomas (19), *IRF2BP2-CDX1* in a mesenchymal chondrosarcoma (20), and *EWSR1-YY1* in a subset of mesotheliomas (21). Except the *ZC3H7-BCOR*, which was detected in a tumor whose karyotype contained two chromosome translocations (19), all the other studies started with malignancies in which the karyotype had a single chromosomal translocation. In hematologic malignancies, likewise, an *NFIA-CBFA2T3* (*NFIA* is located in 1p31) chimeric transcript was found in an acute erythroid leukemia with the translocation t(1;16)(p31;q24) and a FISH-detected split of *CBFA2T3* in 16q24 (22,23). The same approach comparing karyotypic and sequencing data was also used to identify the *ZMYND8-RELA* fusion in a congenital acute erythroid leukemia carrying a t(11;20)(p11;q13) translocation as a sole chromosome aberration (24). In the present study, we applied RNA-Seq methodology to an acute myeloid leukemia with a rather complex karyotype and identified a cryptic *FUS/ERG* fusion gene.

## Case report

### Ethics statement

The study was approved by the Regional Ethics Committee (Regional Komité for Medisinsk Forskningsetikk Sør-Øst, Norge, http://helseforskning.etikkom.no), and written informed consent was obtained from the patient’s parents to publication of the case details.

### Case report

A 2 years and 9 months old girl who had been treated for pneumonia 3 months ago was referred to the local hospital with fever, diarrhea, abdominal pain, reduced general condition, and anemia. Upon admission, she had a hemoglobin value of 9.2 g/dl, thrombocytes of 132×10^9^/l, and signs of cholestasis and pancreatitis (increased pancreas-amylase 418 U/l and lipase 1653 U/l, edematous pancreas on abdominal MRI, some peritoneal cavity fluid). No infectious origin could be found, and investigations for the presence of Epstein-Barr virus, parvovirus B19, hepatitis A, B and C, chlamydia pneumoniae, cytomegalo-virus, and streptococci were negative. After a short period of spontaneous clinical improvement and normalization of liver and pancreatic values, her condition again worsened. Due to recurrent fever and a sore throat, she was started on penicillin. However, she deteriorated, and hemoglobin fell to 7 g/dl. At this point, a bone marrow examination was performed and an increased number of blast-like cells were seen. The patient was then referred to the tertiary care pediatric hematology unit.

Upon admission, the patient was in good clinical shape. She had hypertrophic tonsils without signs of inflammation. There was no pathological enlargement of lymph nodes and no hepatosplenomegaly. Hemoglobin was 7.3 g/dl, the platelet count 222×10^9^/l, and the white blood cell count 6.2×10^9^/l. Lactate dehydrogenase was elevated (914 U/l) but liver parameters were normal. Examination of a bone marrow aspirate revealed 50% myeloid blasts, consistent with acute myeloid leukemia. The myeloid cell lineage of the blasts was confirmed by flow cytometric immunophenotyping showing the expression of the myeloid markers CD13, CD33, CD15, and myeloperoxidase in addition to the aberrant expression of CD7 and CD56. The blasts were positive for the progenitor cell markers CD34, CD117, and CD133, but negative for HLA-DR antigens. Interestingly, a leukemic stem cell population comprising 3% of the total number of cells was also identified. These CD34 brightly positive/CD38 negative leukemic stem cells displayed an abnormal phenotype being negative for HLA-DR antigens but positive for CD7, CD56, and CD123. Molecular genetic analysis did not show any Flt3 internal tandem duplication (ITD) mutation nor were CEPBα mutations seen. As expected, markedly increased levels of Wilms’ tumor (WT) 1 expression were demonstrated.

The girl was started on AML chemotherapy according to the NOPHO-AML 2004 protocol (25) (Nordic Society of Pediatric Hematology and Oncology). She went into remission after the first course (AIET: cytarabine, idarubicin, etoposide, thioguanin). Minimal residual disease by flow cytometry was positive, but <0.1%. This was also the case after the second course (AM: cytarabine, mitoxantrone). However, WT1 levels were normalized. This was also true after the second chemotherapy course (AM: cytarabine, mitoxantrone). Following the third course (HA1M: high dose cytarabine, mitoxantrone), MRD began slowly to increase. After the fourth course (HA2E), the MRD level by flow cytometry was 0.8% indicating relapse, and also the WT1 levels gradually increased. The patient was taken off protocol and re-induced with two courses of FLA (fludarabine, cytarabine). After an initial MRD decrease to 0.4%, it again rose to 3.5% after the second FLA course. Awaiting completion of a bone marrow donor search, the patient is now scheduled for a CloEC course (clofarabine, etoposide, cyclophosphamide) after which the plan is to perform allogenic stem cell transplantation.

### G-banding analysis

Bone marrow cells were cytogenetically investigated by standard methods. Chromosome preparations were made from metaphase cells of a 24-h culture, G-banded using Leishman stain, and karyotyped according to ISCN 2009 guidelines (26).

### Fluorescence in situ hybridization (FISH)

Fluorescence *in situ* hybridization (FISH) was performed on metaphase spreads using the Vysis FUS Break Apart FISH Probe Kit (Abbott Norge Molecular, Snaroya, Norway) and whole painting probes for chromosomes 1, 2, 3, and 16 (Cytocell, BioNordika, Lysaker, Norway). Fluorescent signals were captured and analyzed using the CytoVision system (Leica Biosystems, Newcastle, UK).

### Whole transcriptome sequencing

Total RNA (3 μg) extracted from the patient’s bone marrow at the time of diagnosis was sent for high-throughput paired-end RNA-sequencing to the Norwegian Sequencing Centre at Ullevål Hospital (http://www.sequencing.uio.no). The Illumina software pipeline was used to process image data into raw sequencing data and only sequence reads marked as ‘passed filtering’ were used in the downstream data analysis. A total of 103 million reads were obtained. The FASTQC software was used for quality control of the raw sequence data (http://www.bioinformatics.babraham.ac.uk/projects/fastqc). The software FusionMap was used for the discovery of fusion transcripts (27) (release date 2012-04-16) together with the pre-built Human B37 and RefGene from the FusionMap website (http://www.omicsoft.com/fusionmap).

### PCR analyses

Total RNA (1 μg) was reverse-transcribed in a 20-μl reaction volume using iScript Advanced cDNA Synthesis Kit for RT-qPCR according to the manufacturer’s instructions (Bio-Rad, Oslo, Norway). cDNA corresponding to 50 ng total RNA was used as template in subsequent PCR assays. The 25 μl PCR volume contained 12.5 μl Premix Ex Taq™ DNA Polymerase Hot Start Version (Takara Bio, AH Diagnostics, Oslo, Norway), 1 μl of diluted cDNA, and 0.2 μM of each of the forward and reverse primers. For the detection of the *FUS-ERG* fusion transcript the forward FUS-358F (CAG AGC TCC CAA TCG TCT TAC GG) and the reverse ERG-1163R (CAG GAG CTC CAG GAG GAA CTG C) primers were used. The PCR was run on a C-1000 Thermal cycler (Bio-Rad) with an initial denaturation at 94°C for 30 sec, followed by 35 cycles of 7 sec at 98°C, 30 sec at 60°C, 1 min at 72°C, and a final extension for 5 min at 72°C. PCR products (4 μl) were stained with GelRed (Biotium, VWR International, Oslo, Norway), analyzed by electrophoresis through 1.0% agarose gel, and photographed. The remaining PCR products were purified using the NucleoSpin^®^ Gel and PCR Clean-up kit (Macherey-Nagel, VWR International) and sequenced at GATC Biotech (Germany, http://www.gatc-biotech.com/en/home.html). The BLAST software (http://blast.ncbi.nlm.nih.gov/Blast.cgi) was used for computer analysis of sequence data.

## Results

**Figure 1:**
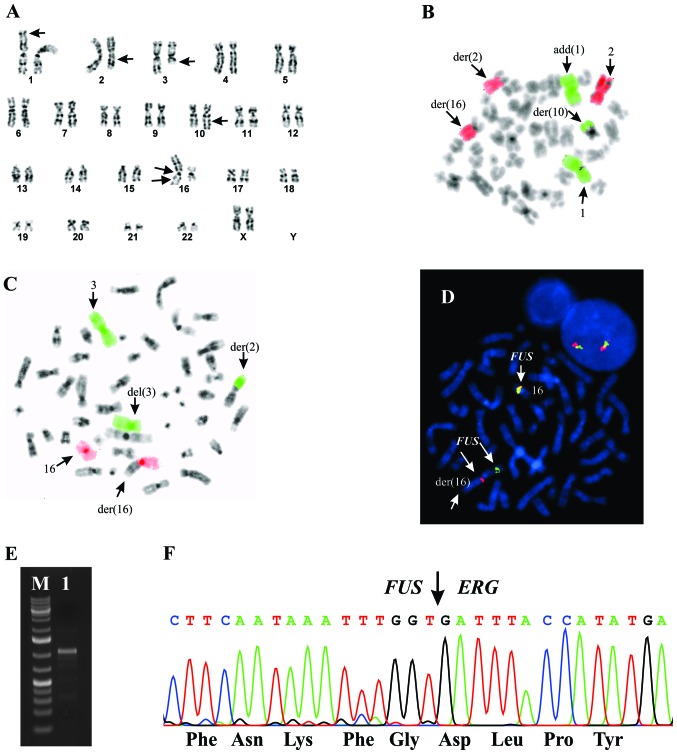
G-banding, FISH, and PCR analyses of the AML. A, G-banded karyotype showing the chromosome aberrations (arrows). B, FISH using WCP for chromosome 1 (green signal) and chromosome 2 (red signal) shows (arrows) the add(1)(p36), der(2)t(2;3)(q21;q21), and der(10)t(1;10)(q32;q24). C, FISH using WCP for chromosomes 3 (green signal) and 16 (red signal) shows (arrows) the der(2)t(2;3)(q21;q21), del(3), and der(16). D, FISH using a *FUS* break apart probe shows (arrows) rearrangement of *FUS* together with the normal chromosome 16. The 5′-part of the *FUS* gene (green probe) has moved to the q arm of the der(16), while the 3′-part of the gene (red probe) remains on 16p11 of the der(16). E, Amplification of *FUS-ERG* fusion cDNA fragments using the primers FUS-358F and ERG-1163R (lane 1). M, 1 kb DNA ladder. F, Partial sequence chromatogram showing the junctions of the *FUS-ERG* chimeric transcript and part of the in-frame coding protein.

The G-banding analysis together with whole chromosome paint FISH yielded the karyotype 46,XX,add(1)(p36),der(2)t(2;3)(q21;q21),del(3)(q21),der(10)t(1;10)(q32;q24),der(16)(2qter--> 2q21::16p11-->16q24::16p11-->16pter)[13]/46,XX[2] (Fig. 1A–C). FISH with a *FUS* break apart probe showed that the *FUS* gene had been rearranged and the 5′-end part of *FUS* (green probe in Fig. 1D) moved to the q arm of the der(16), whereas the 3′-end part of the gene (red probe in Fig. 1D) remained on 16p11 of the der(16).

Using FusionMap on the raw sequencing data obtained by the Norwegian Sequencing Centre, 500 fusion genes were found. The *FUS-ERG* fusion transcript was ranked fourth with 21 seed counts. Because of this and because the chromosome band 16p11 was involved in the complex karyotype (*FUS* maps to chromosome bands 16p11), we decided to investigate further the *FUS-ERG* fusion transcript. PCR and direct sequencing verified the presence of a *FUS-ERG* chimeric transcript in which exon 7 of *FUS* (nt 904 in sequence with accession number NM_004960 version 3) was fused in frame to exon 8 of *ERG* from sub-band 21q22.2 (nt 967 in NM_004449 version 4) (Fig. 1E and F).

## Discussion

The present case of AML had a karyotype with five structural chromosome aberrations, all of which could have generated fusion genes. For example, the *PRDM16* gene in 1p36, which codes for a transcription factor, and the *RPN1* gene in 3q21, which codes for type I integral membrane protein found in the rough endoplasmic reticulum, are rearranged in AML with t(1;3)(p36;q21) (28). Both chromosome bands, 1p36 and 3q21, were seen rearranged in the abnormal karyotype of the present case. Likewise, on chromosome band 16p11, the *FUS* gene was found to be rearranged and fused to *ERG* in a subset of AML with t(16;21)(p11;q22) (1,2), and on 16q24, the *CBFA2T3* gene was found, also in AML, to be a partner gene in the fusions *RUNX1-CBFA2T3* [t(16;21)(q24;q22)], *NFIA-CBFA2T3* [t(1;16)(p31;q24)], and *CBFA2T3-GLIS2* [inv(16)(p13q24)] (22,23,29,30). The aberrations der(2)t(2;3)(q21;q21) and der(10)t(1;10)(q32;q24) could also conceivably generate fusion genes. Screening with FISH for all possibly rearranged genes associated with the present abnormal karyotype would have been laborious and a very time-consuming procedure. We therefore decided to perform RNA-Seq to compare karyotyping and sequencing data and concentrate exclusively on those suggested fusion genes that are found in chromosomal breakpoints. From the 500 fusion genes which were indicated by the RNA-Seq data using the FusionMap algorithm, only *FUS-ERG* showed correspondence with karyotype features (*FUS* maps to 16p11, a chromosome band which was rearranged in the karyotype). Furthermore, the *FUS-ERG* fusion gene has previously been described in a subset AML with t(16;21)(p11;q22) (1,2).

**Figure 2:**
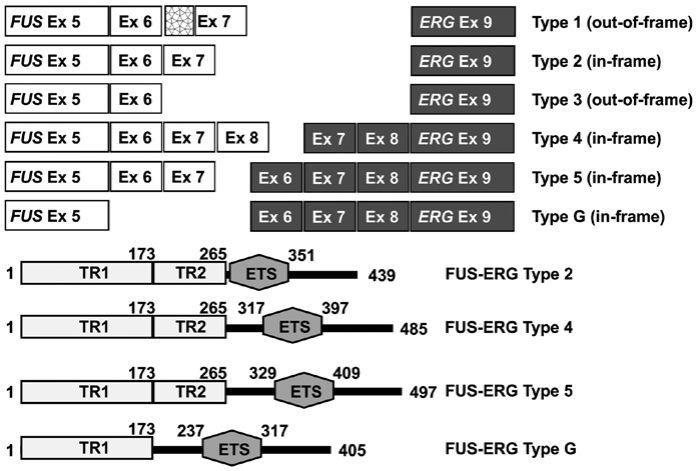
Diagram showing the five known variants of *FUS-ERG* chimeric transcripts found in leukemias and the predicted FUS-ERG proteins of types 2, 4, and 5. All FUS-ERG proteins retain the TR1 and TR2 domains of the transactivation domain of FUS and the ETS DNA binding domain of ERG. For comparison, the *FUS-ERG* chimeric transcript G and the FUS-ERG chimeric protein G are shown. The FUS-ERG protein G retains the TR1 domain of FUS and the ETS DNA binding domain of ERG.

Five different *FUS-ERG* transcript types have been reported in AML (Fig. 2), and three additional types in Ewing tumors (types 4, 5, 8, 9, 31). Types 1 and 3 (named A and C in 4, 8, 9) are out-of-frame transcripts due to the presence of an extra 44 bp sequence of intronic *FUS* material or the absence of 35 bp exonic sequence of *FUS*(4). All other *FUS-ERG* transcript types are in-frame with exons 5, 6, 7 or 8 of *FUS* (according to the reference sequence with accession number NM_004960 version 3) fused with exons 8, 9, 10 or 11 of *ERG* (according to the reference sequence with accession number NM_004449 version 3). Exons 8–11 of *ERG* correspond to exons 6, 7, 8, and 9 of *ERG* in the study by Kong *et al*(4) who used the numeration of *ERG* exons presented by Zucman *et al*(13). For the numbering of *ERG* exons, Zucman *et al*(13) had assumed an identical genomic organization for *ERG* and *FLI1*. All the FUS-ERG isoforms contained the ETS domain which is encoded by exon 11 of *ERG* and the N-terminal transactivation domain of FUS (Fig. 2) (4,9). The N-terminal transactivation domain of FUS can be further subdivided into two independent functional domains, TR1 and TR2. TR1 consists of amino acid residues 1–173 and comprises exons 1–5 of *FUS*, whereas TR2 consists of amino acid residues 174–265 and comprises exons 6 and 7 of *FUS*(32). Deletion studies of the FUS/ERG fusion protein have shown that TR1 is necessary for transformation of mouse fibroblast NIH3T3 cells while TR2 is essential for transformation of the mouse myeloid precursor cell line L-G. This suggests that the TR2 domain is critical for the leukemogenic potential of FUS-ERG fusion protein. In fact, *FUS* exons 1–7 (representing the TR1 and TR2 domains) are always included in AML *FUS/ERG* transcripts, whereas the TR2 domain is sometimes omitted from the fusion gene in solid tumors (4,8).

The *FUS-ERG* transcript found here was of type 5 which has hitherto only been reported in two other cases of childhood leukemia (in a 1-year-old boy and an 8-month-old boy) both diagnosed with precursor B cell ALL (5,31). This fusion transcript codes for a 497-aa FUS-ERG protein which, similar to other AML-related FUS-ERG fusion proteins, would contain both functional domains TR1 and TR2 of the transactivation domain of FUS and the ETS domain of ERG (Fig. 2). It is still not clear whether any biological, let alone clinical, significance can be attributed to the presence of exons 8, 9, and 10 of *ERG* in the FUS-ERG fusion proteins. Future studies should be explicit as to whether exons 8, 9, and 10 of *ERG* were present or not in the chimeric gene; however, given the rarity of this type of transcript, statistically comparable groups of cases with the various potentially important molecular genetic characteristics may not be possible to establish.
